# Job satisfaction and related factors among Iranian intensive care unit nurses

**DOI:** 10.1186/s13104-018-3913-5

**Published:** 2018-11-20

**Authors:** Somayeh Mousazadeh, Shahrzad Yektatalab, Marzieh Momennasab, Soroor Parvizy

**Affiliations:** 1 Amol Faculty of Nursing and Midwifery, Mazandaran University of Medical Science, Sari, Iran; 2 Community Based Psychiatric Care Research Center, Department of Mental Health and Psychiatric Nursing, School of Nursing and Midwifery, Shiraz University of Medical Sciences, Zand Blv, Namazi Square, Shiraz, 71936-13119 Iran; 3 Department of Nursing, School of Nursing and Midwifery, Shiraz University of Medical Sciences, Shiraz, Iran; 4 Department of Pediatric Nursing, Nursing and Midwifery Faculty, Center for Educational Research in Medical Sciences (CERMS), Iran University of Medical Sciences, Tehran, Iran

**Keywords:** Job satisfaction, Demographic characteristics, Nurses, ICU

## Abstract

**Objectives:**

The aim of this study is to determine the levels of job satisfaction and to collect information about the factors affecting job satisfaction of Iranian ICU hospital nurses.

**Results:**

The participants included 124 nurses working in the ICU section of hospitals in the city of Amol in Iran, who were selected by census method. The instruments for gathering the information included Demographic Information Questionnaire and also the Minnesota Satisfaction Questionnaire. The results revealed that the average score of job satisfaction among ICU nurses was 2.50 ± 0.51. Also job satisfaction among women was higher than men (P = 0.03, t = 0.4). One way analysis of variance showed a significant relation between job satisfaction level with employment status and overtime work. Also older nurses had higher levels of job satisfaction. Hospital directors and managers, can use the results of this study in order to have a deeper understanding of job satisfaction among nurses, and the factors affecting it.

## Introduction

Job satisfaction is a series of positive feelings and attitudes of a person toward his or her job, and if these feelings fade away, a person may quit his or her job. This concept also refers to the joy and excitement of the person resulting from his or her evaluation and attitude toward his or her job and the emotional reactions concerning the job [1]. Various authors have different definitions of job satisfaction. According to the Armstrong, the definition of job satisfaction is the attitude and feelings people have about their work. Positive and favorable attitudes towards the job indicate job satisfaction [2]. According to Kaliski definition, Job satisfaction is a worker’s sense of achievement and success on the job. It is the key ingredient that leads to recognition, income, promotion, and the achievement of other goals that lead to a feeling of fulfillment [3].

An unfavorable working condition for a nurse may reduce the satisfaction of a nurse, and therefore will interfere with efforts to improve patients’ satisfaction because high quality nursing care is usually a result of high levels of job satisfaction [4]. In today’s world, the importance of evaluating the patient satisfaction as one of the main criteria to determine the healthcare quality is evident [5] and nursing care is one of the main factors of patient satisfaction regarding the quality of the healthcare [6, 7].

Tension and job dissatisfaction among nurses can threaten their physical and emotional health, and also affect the quality of their lives and may become an obstacle against achieving personal and social development, which can also affect the goals of an organization [8]. Also the finding of study by Karasek et al. [9], was show, the combination of low decision latitude and heavy job demands which is associated with mental strain.

Nurses working in a hospital, especially in the ICU section, need to deal with different factors which can affect their job satisfaction. Some of these factors include: demographic characteristics, job specifications, working environment, and the ability to effectively be in charge of working issue [10]. Job dissatisfaction among nurses in the ICU section not only causes heavy financial losses, but it can have negative effects on nurses as well as the well-being of the patients. Nurses working in these sections are usually responsible for the constant monitoring of the patient conditions, their medication, interpreting and working with different machines and changes in the patient’s behavior may influence the nurses’ performance and also affect their job satisfaction. Another evident characteristic of ICU nurses is their exact and delicate concentration on the patients’ conditions which makes the ICU care stressful and affects the nurses’ health [11]. Thus, it is very important to determine which factors can affect the job satisfaction of the nurses working in ICU. Also examining the effective factors on job satisfaction is an intriguing issue for researchers. However, only a scarce number of studies have been done in Asian countries [10]. Hashemian et al. study evaluated the stress among Iranian nurses and revealed that marital status, work shift, and years of experience as independent factors of stress [12]. There is a dearth of empirical evidence about factors of stress among Iranian nurses which effect on Job satisfaction.

Hence, due to immense cultural changes, the findings of the studies conducted in Western countries cannot be quite applicable in Eastern countries such as Iran. Thus, as a result of reviewing the literature and understanding the scarcity of studies regarding job satisfaction and its affecting factors among Iranian ICU nurses, the present study was done in order to examining the job satisfaction and its related factors among ICU nurses.

## Main text

### Methods

This study uses a cross-sectional research designed to determine the levels of job satisfaction and also to collect information about the factors affecting job satisfaction among Iranian hospital nurses.

The sample of this study included 124 nurses working in the general, poisoning, and trauma ICU of four hospitals affiliated with Mazandaran Medical University who were included in the study by census method. The criteria for being included in the study were: a minimum of 1-year experience of working in the ICU section; having an Associates’ Degree, a BA or higher levels of education in Nursing; and also the willingness to participate in the study. Incomplete questionnaires were considered as a criterion of exclusion.

Since the Persian version of this instrument has already been used in another study, its reliability and validity were approved in that study. In a study done by Jaafari et al. the reliability of the questionnaire was calculated through test–retest process with a correlation coefficient of 0.78 and its content validity was approved by a team of specialists [13]. In another study the Cronbach alpha coefficient was 0.81 [14] and in a study conducted by Weiss et al. reported a Cronbach alpha of 0.70 [15].

The study is approved by ethical committee of Shiraz University of Medical Sciences. After obtaining permission from the hospital directors, the researcher went to the ICU and introduced herself and got a written consent letter of awareness. Next, she described the objectives of her study and explained how the nurses could fill out the questionnaires. If the nurses were willing to participate in the study, they were given the questionnaires and they were assured to remain anonymous and also assured that if they did not participate in the study, they would not get any negative points in any way. In order to conduct this study, all the nurses working in the ICU sections were considered as the sample. At the end of the data collection, SPSS software (version 19) was used in descriptive analysis (frequency, mean, and standard deviation) and inferential statistics (t-test and one-way ANOVA). In all the tests, a significance level of 5% was defined. In order to test the normalization of the variables the Kolmogorov–Smirnov statistical test (K–S test) was used.

### Results

The results of the K–S test (P = 0.69) showed that the data had normal distribution. The participants included 124 nurses working in the ICU sections. Table 1 shows the demographic characteristics of the participants of this study. The result showed that most of the participants were female (71%) and married (82.3%). The age range of the participants was between 20 and 50 years old. Also 33.9% of the participants were aged between 26 and 30. Many nurses (36.3%) had a job experience between 1 and 5 years. And most of them (98.4%) had a BA in Nursing. 42.7% of the participants were working based on a contract and 92.7% of them had rotational shifts. 51.8% of the participants had between 50 and 100 h of extra work (overtime working) in a month (Table 1).

**Table 1:** Demographic characteristics for the sample (n = 124)

Demographic characteristics	N	%
Gender		
Male	36	29
Female	88	71
Age (year)		
20–25	15	12.1
26–30	42	33.9
31–35	31	25
36–40	18	14.5
41–45	12	9.7
46–60	6	4.8
Marital status		
Single	22	17.7
Married	102	82.3
Education level		
Associate’s degree	1	0.8
BA	122	98.4
MA	1	0.8
Years of employment (year)		
1–5	45	36.3
6–10	42	33.9
11–15	28	22.6
16–20	8	6.5
21–25	1	0.8
Employment status		
Contract	20	16
Commission	53	42.7
Employed	30	24.2
Company	8	6.5
Trainee	13	10.5
Working shifts		
Morning	5	4
Evening	2	1.6
Rotational	115	92.7
Night	2	1.6
Monthly overtime work (hours)		
Less than 50	42	33.0
Between 50 and 100	72	58.1
More than 100	10	8.1

The results showed that the average score of job satisfaction among nurses was 2.50 ± 0.51. The independent t-test showed that there was no significant relationship between job satisfaction and marital status. The results also indicated no significant relationship between job satisfaction of ICU nurses and their education level, working shifts, and working experience. The results indicated that there was a significant difference between job satisfaction of male and female nurses and the female’s job satisfaction was more than males (P = 0.03, t = 0.4).

The one-way ANOVA showed a significant difference between job satisfaction and employment, also the post hoc test (LSD) showed that job satisfaction of the trainees was less than the others (P ≤ 0.01).

There was a significant relationship between job satisfaction levels of nurses and the amount of overtime work in a month. Based on the post hoc LSD test, it was revealed that nurses who had over 100 h of monthly overwork had a more job satisfaction compared to nurses with less than 50 h of overwork (P = 0.01) and compared to nurses with 50–100 h (P ≤ 0.05). The results also indicated that as the nurses get older, they have more job satisfaction and older nurses had more job satisfaction (Table 2).

**Table 2:** A comparison of job satisfaction among nurses with three variables: age, employment status and monthly overtime work (hours)

Demographic characteristics	P value	F	*df*
Age (year)	0.03	2.97	125
Employment status	0.04	2.55	123
Monthly overtime (hours)	0.02	3.68	123

### Discussion

The findings of the present research indicated that levels of job satisfaction of the subjects under study was an average level. Similarly, the findings of a study done by Mehrdad et al. in Iran showed that 47.9% of the nurses were satisfied with their jobs [16]. Also a study conducted by Mastaneh et al. showed that the general job satisfaction of Iranian nurses was at an average level [17]. However, a study conducted in the CCU sections of Shanghai hospitals by Liu et al. showed that more than half of the nurses were dissatisfied with their jobs [18]. Atefi et al. conducted a study in the city of Mashhad (Iran) and reported that only 28.7% of nurses were satisfied with their jobs [19]. Such diversity in the results can be explained by the cultural situations of the society and the facilities that organizations provide for the nurses.

Regarding the relationship between gender and job satisfaction level, there are controversial results. The results of the present research showed a significant difference between job satisfaction levels regarding gender: job satisfaction among women is more than men. A study conducted by Asghari et al. in the city of Rasht (Iran) showed that men had 13% higher job satisfaction compared to women [20]. Yet, the results of other studies showed no significant relationship between job satisfaction and gender [21]. Considering the Iranian culture, women tend to have more willingness in achieving financial and social independence which can explain the higher levels of job satisfaction among women. Furthermore, in Iranian families, men are considered the breadwinner of the family and fulfilling the financial needs of the family mostly depends on men, and these financial needs are not completely fulfilled by their jobs; this may also account for men’s lower levels of job satisfaction.

In the present study, job satisfaction of trainees was less than job satisfaction of employed, contract and company nurses. Another study showed that job satisfaction of contract nurses was more than others [20]. The reason for the low job satisfaction among trainees in Iran might be due to the fact that they are generally amateurs with low levels of experience; they have recently graduated from universities and have very little clinical and professional experience. They learn things during their education which may not meet clinical needs, therefore when these people face the actual situation, they have a feeling of confusion and suppression which can lead to job dissatisfaction. Nonetheless, as time passes, they gain more experience or they get used to the present situation, and they learn the needed skills, so they will be more satisfied.

Based on the findings of the present research the people who more hours of extra had hours during the month had a higher job satisfaction compared to the auditors. More extra hours in a month means a higher salary and income is one of the most important factors in creating a feeling of job satisfaction [22, 23]. In this regards, a study by Malik et al. showed that income had a significant influence on job satisfaction [24]. Another study by Mastaneh et al. conducted on Iranian nurses showed that the amount of the income and facilities had significant effects on people’s job satisfaction [17].

The results revealed that older nurses had higher levels of job satisfaction compared to their younger counterparts. Also Asghari et al. showed that most participants who were satisfied with their jobs, were in the age range of 41 and above [20]. But, the results of a research conducted by Mastaneh et al. showed that job satisfaction among young people was more [17]. The satisfaction of young personnel when entering the system is generally high; however, when their needs are not met, most of them will dissatisfied. Also as people become older and more experienced, their expectations will reduce to practical and real-life levels; as a result, their expectations will be somehow achievable, which can lead to an increase in their job satisfaction.

Job dissatisfaction among nurses is one of the most important factors in quitting their jobs, which increases the need for more nurses, and in turn increases the workload of the current nurses, leading to their dissatisfaction. Therefore, nursing directors and managers need to determine the effective factors on nurses’ job satisfaction and they need to control the factors which decrease their satisfaction and take some measures in order to improve their job satisfaction levels and also they need to consider demographic and job factors in order to periodically evaluate the effectiveness of their measures to improve job satisfaction.

## Limitations

This study had some limitations; for example, sampling was limited to four hospitals. It is recommended that future studies examine a larger sample and more hospitals. Another limitation of the study was the fact that heavy workload of the nurses and their physical and emotional tiredness when answering the questionnaires could have affected the results. However, some gifts were bought and given to reduce the negativity of this factor.
